# Supplementary material for: Pregnancy complications recur independently of maternal vascular malperfusion lesions
Source: PLoS One. 2020 Feb 6;15(2):e0228664. doi: 10.1371/journal.pone.0228664 (PMC7004354; doi:10.1371/journal.pone.0228664)
Supplement: S1 Table — (DOCX) [file pone.0228664.s001.docx]

Supplemental Table 1. Characteristics of pregnancies with and without missing placental data, including only pregnancies at 24 weeks or later and excluding 3 institutions where placental pathology data were available for less than 75% percent of pregnancies. Means are ± standard error.

|  | MVM available | MVM not available | P-value |
| --- | --- | --- | --- |
| N | 11753 (81.4%) | 2686 (18.6%) |  |
| Maternal age | 24.91 ± 0.05 | 25.27 ± 0.10 | 0.002 |
| Maternal race |  |  | 0.0005 |
| White | 6861 (80.5%) | 1666 (19.5%) |  |
| Black | 4892 (82.8%) | 1020 (17.2%) |  |
| Pre-pregnancy BMI | 22.65 ± 0.04 | 22.88 ± 0.09 | 0.02 |
| Tobacco use |  |  | 0.30 |
| No | 5495 (81.4%) | 1260 (18.6%) |  |
| Yes | 6150 (82.0%) | 1348 (18.0%) |  |
| Gestational age at delivery | 39.06 ± 0.03 | 38.64 ± 0.06 | 0.0001 |
| Birthweight | 3172 ± 5 | 3140 ± 12 | 0.01 |
| Preeclampsia |  |  | 0.95 |
| No | 10834 (82.9%) | 2230 (17.1%) |  |
| Yes | 308 (82.8%) | 64 (17.2%) |  |
| SGA |  |  | 0.13 |
| No | 10620 (82.2%) | 2302 (17.8%) |  |
| Yes | 1058 (80.5%) | 256 (19.5%) |  |
| Preterm |  |  | 0.0001 |
| No | 9600 (82.4%) | 2058 (17.6%) |  |
| Yes | 2153 (77.4%) | 628 (22.6%) |  |
| Survival |  |  | 0.0001 |
| Fetal death | 131 (54.1%) | 111 (45.9%) |  |
| Death before 120 days | 236 (78.2%) | 66 (21.8%) |  |
| Survival past 120 days | 11386 (81.9%) | 2509 (18.1%) |  |
