# Supplementary material for: Pregnancy complications recur independently of maternal vascular malperfusion lesions
Source: PLoS One. 2020 Feb 6;15(2):e0228664. doi: 10.1371/journal.pone.0228664 (PMC7004354; doi:10.1371/journal.pone.0228664)
Supplement: S2 Table — (DOCX) [file pone.0228664.s002.docx]

Supplemental Table 2. Recurrence of outcomes between pregnancies, restricting analyses to women not showing MVM lesions in either pregnancy.

|  | Excluding cases with MVM_narrow_ | | | Excluding cases with MVM_broad_ | | |
| --- | --- | --- | --- | --- | --- | --- |
|  | Adjusted odds ratio^1^ | P-value | Other significant terms in model | Adjusted odds ratio^1^ | P-value | Other significant terms in model |
|  | (95% CI) |  |  | (95% CI) |  |  |
| Preeclampsia | 4.9 (2.6 – 9.6) | 0.0001 | Race (P = 0.0005); BMI (P = 0.0008) | 5.2 (2.3 – 11.9) | 0.0001 | Race (P = 0.004) |
|  |  |  |  |  |  |  |
| SGA | 4.1 (3.0 – 5.5) | 0.0001 | Gestational age (P = 0.02); Smoking (P < 0.0001) | 4.6 (3.2 – 6.5) | 0.0001 | Smoking (P < 0.0001) |
|  |  |  |  |  |  |  |
| Prematurity |  | 0.0001 | Race (P < 0.0001); BMI (P = 0.03) |  | 0.0001 | Race (P = 0.005); BMI (P = 0.04) |
| Extreme | 5.8 (3.0 – 11.2) |  |  | 4.0 (0.8 – 20.0) |  |  |
| Very | 3.9 (2.7 – 5.7) |  |  | 8.7 (3.7 – 20.6) |  |  |
| Moderate | 3.5 (2.8 – 4.3) |  |  | 3.3 (2.2 – 5.0) |  |  |
| Survival |  | 0.17 | Gestational age (P < 0.0001) |  | 0.96 | Gestational age (P < 0.0001) |
| Fetal death | 3.4 (0.9 – 12.4) |  |  | Non-estimable |  |  |
| Death before 120 days | 0.9 (0.2 – 3.8) |  |  | 0.8 (0.1 – 5.0) |  |  |
| Apgar score at 1 minute |  | 0.02 | Gestational age (P < 0.0001) |  | 0.21 | Gestational age (P < 0.0001) |
| 0-3 | 1.4 (1.0 – 2.1) |  |  | 1.4 (0.8 – 2.5) |  |  |
| 4-6 | 1.4 (1.1 – 1.7) |  |  | 1.3 (0.9 – 1.7) |  |  |
| Apgar score at 5 minutes |  | 0.006 | Gestational age (P < 0.0001) |  | 0.58 | Gestational age (P < 0.0001); Race (P = 0.03) |
| 0-3 | 3.6 (1.3 – 9.8) |  |  | 2.2 (0.5 – 10.1) |  |  |
| 4-6 | 2.4 (1.1 – 5.4) |  |  | 1.3 (0.3 – 5.7) |  |  |

^1^ Adjusted odds ratios are from logistic regression including interpregnancy interval, gestational age, maternal race, maternal BMI and smoking. Analyses of prematurity and spontaneous abortion before 20 weeks did not include gestational age.
