# Supplementary material for: Pregnancy complications recur independently of maternal vascular malperfusion lesions
Source: PLoS One. 2020 Feb 6;15(2):e0228664. doi: 10.1371/journal.pone.0228664 (PMC7004354; doi:10.1371/journal.pone.0228664)
Supplement: S4 Table — (DOCX) [file pone.0228664.s004.docx]

Supplemental Table 4. Adjusted odds ratios for all covariates in analyses of effects of previous spontaneous abortions on pregnancy outcomes.

|  | Previous spontaneous abortions | | Interpregnancy interval | | Gestational age | | Maternal age | | Maternal race | | Maternal BMI | | Smoking | |
| --- | --- | --- | --- | --- | --- | --- | --- | --- | --- | --- | --- | --- | --- | --- |
|  | aOR^1^ | P-value | aOR^1^ | P-value | aOR^1^ | P-value | aOR^1^ | P-value | aOR^1^ | P-value | aOR^1^ | P-value | aOR^1^ | P-value |
| MVM_narrow_  (Yes vs No) | 2.2 | 0.02 | 1.6  1.4 | 0.04 | 0.9 | 0.0001 | 1.0 | 0.47 | 1.1 | 0.47 | 1.4  0.8  0.7 | 0.09 | 1.3 | 0.16 |
| MVM_broad_ (Yes vs No) | 0.9 | 0.51 | 1.1  1.2 | 0.11 | 0.7 | 0.0001 | 1.0 | 0.91 | 1.1 | 0.06 | 0.9  1.1  0.8 | 0.34 | 1.0 | 0.53 |
| Preeclampsia  (Yes vs No) | 1.1 | 0.72 | 1.1  1.1 | 0.82 | 0.96 | 0.03 | 1.04 | 0.001 | 0.5 | 0.0001 | 0.9  1.6  3.0 | 0.0001 | 0.7 | 0.03 |
| SGA  (Yes vs No) | 2.4 | 0.0001 | 1.1  1.0 | 0.52 | 1.04 | 0.008 | 1.0 | 0.004 | 0.9 | 0.22 | 1.2  0.6  0.8 | 0.0001 | 2.6 | 0.0001 |
| Prematurity |  | 0.0001 |  | 0.59 |  | --- |  | 0.02 |  | 0.0001 |  | 0.0001 |  | 0.0001 |
| (Extreme vs Term) | 5.1 |  | 1.0  1.1 |  | --- |  | 0.97 |  | 0.3 |  | 1.4  0.4  1.0 |  | 1.2 |  |
| (Very vs Term) | 2.2 |  | 1.2  0.8 |  | --- |  | 0.98 |  | 0.2 |  | 1.7  0.7  0.9 |  | 1.6 |  |
| (Moderate vs Term) | 1.3 |  | 1.1  1.1 |  | --- |  | 0.99 |  | 0.4 |  | 1.5  0.8  0.9 |  | 1.3 |  |
| Spontaneous abortion before 20 weeks  (Yes vs No) | 2.1 | 0.005 | 2.1  1.1 | 0.0002 | --- | --- | 1.1 | 0.0001 | 1.3 | 0.15 | 1.6  0.9  1.6 | 0.03 | 1.4 | 0.05 |
| Survival |  | 0.0001 |  | 0.01 |  | 0.0001 |  | 0.06 |  | 0.006 |  | 0.44 |  | 0.97 |
| (Fetal death vs Survival past 120 days) | 4.6 |  | 0.7  2.0 |  | 0.7 |  | 1.04 |  | 1.5 |  | 0.9  0.9  1.2 |  | 1.0 |  |
| (Death before 120 days vs Survival past 120 days) | 1.3 |  | 1.2  0.8 |  | 0.7 |  | 0.98 |  | 1.6 |  | 1.3  1.1  0.4 |  | 1.0 |  |
| Apgar score at 1 minute |  | 0.02 |  | 0.002 |  | 0.0001 |  | 0.03 |  | 0.03 |  | 0.13 |  | 0.52 |
| (0-3 vs 7-10) | 1.8 |  | 1.2  1.9 |  | 0.87 |  | 1.02 |  | 1.2 |  | 1.3  1.1  1.7 |  | 1.0 |  |
| (4-5 vs 7-10) | 1.5 |  | 1.0  1.3 |  | 0.95 |  | 1.02 |  | 1.2 |  | 1.0  1.0  1.0 |  | 0.9 |  |
| Apgar score at 5 minutes |  | 0.0007 |  | 0.02 |  | 0.0001 |  | 0.27 |  | 0.41 |  | 0.79 |  | 0.13 |
| (0-3 vs 7-10) | 4.5 |  | 1.0  1.7 |  | 0.8 |  | 0.97 |  | 1.2 |  | 1.0  0.9  1.3 |  | 1.3 |  |
| (4-5 vs 7-10) | 0.6 |  | 1.5  1.8 |  | 0.9 |  | 1.01 |  | 0.9 |  | 0.9  1.2  1.4 |  | 1.2 |  |

^1^ All odds ratios were calculated as the odds of the adverse outcome relative to the odds of the best outcome where there were multiple levels of adverse outcomes. For interpregnancy interval, 2 odds ratios are presented: short interval (less than 18 months) vs medium interval (18 to 59 months, inclusive), and long interval (greater than 59 months) vs medium interval. For maternal race, odds ratios are for white vs black. For maternal BMI, 3 odds ratios are presented: underweight vs normal, overweight vs normal, and obese vs normal. For smoking, odds ratios are for smoking vs not smoking.
