# Supplementary material for: Pregnancy complications recur independently of maternal vascular malperfusion lesions
Source: PLoS One. 2020 Feb 6;15(2):e0228664. doi: 10.1371/journal.pone.0228664 (PMC7004354; doi:10.1371/journal.pone.0228664)
Supplement: Attachment — Submitted filename: PLoS ONE Response to Reviews.docx [file pone.0228664.s005.docx]

Dear Dr. Spradley,

Thank you very much for the encouraging response to our manuscript (PONE-D-19-32724), and to the reviewer for their constructive comments. Below, we have outlined our responses to each of the comments, with the reviewer’s comments in italics.

Sincerely,

Julian

**Journal requirements**

*1. Please ensure that your manuscript meets PLOS ONE's style requirements, including those for file naming.*

We have revised the title page to be consistent with PLOS ONE style. We have also revised the names of the supplementary files, and how they are cited within the manuscript.

*2. Please note that all PLOS journals ask authors to adhere to our policies for sharing of data and materials: https://journals.plos.org/plosone/s/data-availability. According to PLOS ONE’s Data Availability policy, we require that the minimal dataset underlying results reported in the submission must be made immediately and freely available at the time of publication. As such, please remove any instances of 'unpublished data' or 'data not shown' in your manuscript and replace these with either the relevant data (in the form of additional figures, tables or descriptive text, as appropriate), a citation to where the data can be found, or remove altogether any statements supported by data not presented in the manuscript.*

We removed 3 statements with “results not shown”. All three cases stated that similar results were obtained when analyses included institution as a covariate. Since institution does not appear to be an important covariate, we have not included these results.

*3. We noticed you have some minor occurrence(s) of overlapping text with the following previous publication(s), which needs to be addressed:*

*https://doi.org/10.1016/j.placenta.2019.01.012*

[*https://doi.org/10.1177%2F1093526619852871*](https://doi.org/10.1177%2F1093526619852871)

*In your revision ensure you cite all your sources (including your own works), and quote or rephrase any duplicated text outside the Methods section. Further consideration is dependent on these concerns being addressed.*

The first publication listed was authored by one of us (JKC) and describes the same dataset, and so there is some overlapping text in the Methods section. In the revised version, we have cited this previous publication at the beginning of the duplicated text.

We have removed the text associated with the second publication. However, we note that in the case the second publication, the overlapping text was due to the description of a number of specific diagnoses and lesions which could not be rephrased, i.e., the repetition of text was not inappropriate. We have confirmed this with Sarah Mills in the Editorial Office.

**Review Comments to the Author**

**Reviewer #1**

*1. The MVM broad category is fundamentally flawed. The narrow category combining histologic MVM with decreased placental weight is justified, but there are many other placental histologies associated with decreased placental weight (e.g. VUE, maternal floor infarction, and severe examples of fetal vascular malperfusion and chronic abruption) just as there are many small placentas without any specific histologic changes. I suggest that the MVM broad category be split into two subgroups (1) decreased placental weight NOS and (2) placentas showing histologic MVM with weights greater than 10th percentile. If this decreases the power to find an association, an alternative MVM broad group would be any histologic MVM irrespective of placental weight.*

We agree with the reviewer’s concerns regarding the broad category. We originally included this category because it was used by a previous publication, and we wanted to allow comparison of different approaches. However, upon reviewing the literature, we can only find one publication using the “broad” approach defined in this way. We have therefore replaced this category with the “alternate” group proposed by the reviewer (i.e., any MVM histology, regardless of placental weight), which is also an approach that has been used previously by others (as cited in revised version). We have not used the subgroups suggested, as the purpose of these analyses is not to compare mutually exclusive groups of pregnancies with each other (low placental weight with no MVM lesion vs. low placental weight with MVM lesion vs. normal placental weight with MVM lesion), but rather to compare different approaches to categorizing MVM lesions that have been used in the literature.

Reanalysis using the new MVM broad category (any MVM lesion, irrespective of placental weight) yields very similar results to our previous MVM broad category, although the prevalence of MVM lesions is somewhat lower (because small placentas with no histological lesions are no longer considered to show MVM lesions).

*2. Abstract line 36: Shouldn't the word "no" be deleted?*

It has been deleted, thanks.

*3. Line 152: I do not understand how the last two words "for 3865" apply to this sentence.*

Text has been reworded.

*4. Lines 158-165 and lines 236-242: These sections address the clinical entity of "recurrent pregnancy loss or miscarriage (3 or more losses)", not the less stringent "previous spontaneous abortion" which would be one or more losses.*

We have used the term “previous spontaneous abortions” rather than “recurrent pregnancy loss” because the latter term is often defined as 3 or more consecutive losses. We only had information on total losses, not consecutive losses.

*5. Lines 263-273: It is not clear whether adjusting for MVM used the narrow or broad definition.*

This has been clarified.

*6. Line 316: The term "recurrent placental pathology" is inaccurate. Should be "recurrent MVM" since there are many other placental pathologies, such as VUE, fetal vascular malperfusion, and others, that cause the same outcomes, can recur, and are associated with small placentas.*

Text revised.

*7. Lines 326-327: Reference 50 addresses recurrent preeclampsia, not adverse outcomes.*

This sentence has been removed (please see response to following comments).

*8. Lines 332-333: There are actually many papers documenting specific patterns of placental pathology in women with recurrent adverse outcomes ( "multiple complicated pregnancies").*

We agree, and have removed this paragraph. Upon reflection, the references that we cite earlier in the Discussion provide better context for our study than case studies.

*9. Lines 347-351: In this section I actually believe that the authors are underestimating the quality of the original data. I have discussed at length the histopathologic coding of the Collaborative Project with its main study pathologist and I would argue that very little has changed since that time. Differences would be changes in terminology, as the authors discuss, and probably some increased sensitivity for detecting mild lesions. Specifically, I do not think that distal villous hypoplasia would have been missed at that time.*

We agree regarding the value and quality of the data, and sought to be conservative in considering the limitations of our study. We have moderated this paragraph in response to the reviewer’s comments. The term “distal villous hypoplasia” is not used in the pathology coding of the dataset, and it is not clear what terms in the NCPP dataset would correspond closely to this lesion, but of course during pathological assessment, it may have been noted and described in other terms.
