# Supplementary material for: Pregnancy complications recur independently of maternal vascular malperfusion lesions
Source: PLoS One. 2020 Feb 6;15(2):e0228664. doi: 10.1371/journal.pone.0228664 (PMC7004354; doi:10.1371/journal.pone.0228664)
Supplement: Attachment — Submitted filename: PLoS ONE Response to Reviews 2nd round.docx [file pone.0228664.s006.docx]

Dear Dr. Spradley,

Thanks very much to you and to the reviewer for your prompt response regarding our revised manuscript (PONE-D-19-32724). Below, we have outlined our responses to each of the comments, with the reviewer’s comments in italics.

Sincerely,

Julian

Reviewers' comments:

*Reviewer #1: The authors have substantially improved the manuscript and demonstate that MVM, as defined by 1960's criteria, is not the predominant reason for recurrent adverse outcomes in subjects with MVM in an index pregnancy. Nevertheless, I am not sure that it should be concluded that MVM has no significant recurrence risk.*

We have modified this conclusion (described further below).

*1. Why is MVM recurrence risk adjusted for gestational age (GA)? MVM can occur at all gestational ages and there is no a priori reason to assume that GA would be a confounder.*

There are two reasons why gestational age was included in the analysis of recurrence. Firstly, we used the same set of covariates for all analyses for consistency and objectivity, i.e., to avoid analysing the data in different ways and then having to make subjective decisions regarding which analysis to use for each outcome. In the case of MVM, we thought that gestational age might influence the probability of MVM diagnosis (e.g., if some lesions were more apparent later in pregnancy, or if some lesions only develop later in pregnancy). As it turns out, gestational age is a highly significant covariate.

*2. Odds ratios are consistently elevated for all comparisons of MVM recurrence in Table 2, adjusted and unadjusted. Recurrence is significant before adjustment for GA in the MVM broad category and borderline significant even after adjustment. The lack of significance for the MVM narrow category might be attributed to small numbers (only 2 subjects with recurrence).*

We have revised the text to acknowledge this (in some cases reverting to text in the original manuscript).

*3. There is no a priori reason to restrict recurrence to the previous MVM category. The modifiers are continuous not categorical. In other words, recurrence for MVM narrow should include both broad and narrow MVM and recurrence for MVM broad should include both broad and narrow MVM.*

The two categories (MVM broad and MVM narrow) are two different criteria for assessing the presence/ absence of the same pathological entity, MVM. We performed analyses with both sets of criteria to allow comparison of these two criteria because some studies have used one approach while others have used the other. Thus, if one believes that MVM narrow provides a better assessment of the presence of MVM, then it wouldn’t make sense to consider the MVM narrow pathology to have recurred if there was MVM broad (but not narrow) in the previous pregnancy and MVM narrow in the subsequent pregnancy. The MVM narrow cases are a subset of the MVM broad cases (i.e., if a pregnancy is categorized as having MVM using the narrow criteria, it will also be so categorized with MVM broad). Therefore, the analysis of recurrence of MVM broad lesions does include MVM narrow lesions in the previous pregnancy.

*I would ask if the authors might accept that MVM has a recurrence rate, albeit small compared to other factors in explaining subsequent adverse outcomes.*

The revisions in response to comment 2 above acknowledge the recurrence of MVM broad lesions.
